# Hepatitis C Prevalence and Management Among Patients Receiving Opioid Substitution Treatment in General Practice in Ireland: Baseline Data from a Feasibility Study

**DOI:** 10.2196/10313

**Published:** 2018-12-19

**Authors:** Ross Murtagh, Davina Swan, Eileen O'Connor, Geoff McCombe, John S Lambert, Gordana Avramovic, Walter Cullen

**Affiliations:** 1 University College Dublin Dublin Ireland; 2 Mater Misericordiae University Hospital Dublin Ireland

**Keywords:** hepatitis C, integrated HCV care, people who inject drugs, primary care

## Abstract

**Background:**

Hepatitis C virus (HCV) infection is a major cause of chronic liver disease and death. Injection drug use is now one of the main routes of transmission of HCV in Ireland and globally with an estimated 80% new infections occurring among people who inject drugs (PWID).

**Objective:**

We aimed to examine whether patients receiving opioid substitution therapy in primary care practices in Ireland were receiving guideline-adherent care regarding HCV screening. Ireland has developed a model of care for delivering opioid substitution treatment in the primary care setting. We conducted this study given the shift of providing care for PWID from secondary to primary care settings, in light of current guidelines aimed at scaling up interventions to reduce chronic HCV infection and associated mortality.

**Methods:**

We included baseline data from the Dublin site of the Heplink study, a feasibility study focusing on developing complex interventions to enhance community-based HCV treatment and improve the HCV care pathway between primary and secondary care. We recruited 14 opioid substitution treatment-prescribing general practices that employed the administration of opioid substitution therapy from the professional networks and databases of members of the research consortium. A standardized nonprobability sampling framework was used to identify 10 patients from each practice to participate in the study. Patients were eligible if aged ≥18 years, on opioid substitution treatment, and attending the practice for any reason during the recruitment period. The baseline data were collected from the clinical records of participating patients. We collected and analyzed data on demographic characteristics, care processes and outcomes regarding HCV and other blood-borne viruses, urinalysis test results, alcohol use disorders, chronic illness, and health service utilization. We examined whether patients received care concordant with guidelines related to HCV screening and care.

**Results:**

The baseline data were collected from clinical records of 134 patients; 72.2% (96/134) were males; (mean age 43, SD 7.6; range 27-71 years); 94.8% (127/134) of patients had been tested for anti-HCV antibody in their lifetime; of those, 77.9% (99/127) tested positive. Then, 83.6% (112/134) of patients had received an HIV antibody test in their lifetime; of those, 6.3% (7/112) tested HIV positive. Moreover, 66.4% (89/134) of patients had been tested for hepatitis B virus in their lifetime and 8% (7/89) of those were positive. In the 12 months before the study, 30.6% (41/134) of patients were asked about their alcohol use by their general practitioner, 6.0% (8/134) received a brief intervention, and 2.2% (3/134) were referred to a specialist addiction or alcohol treatment service.

**Conclusions:**

With general practice and primary care playing an increased role in HCV care, this study highlights the importance of prioritizing the development and evaluation of real-world clinical solutions that support patients from diagnosis to treatment completion.

## Introduction

### Background

Hepatitis C virus (HCV) infection is associated with considerable morbidity and public health burden globally. Worldwide, it is estimated that approximately 115 million people (1.6% of the world’s population) have been infected with HCV, two-thirds of the infections being chronic [1]. The number of people infected with HCV infection in the European Union (EU) and European Economic Area is estimated at 5.6 million [2].

The major burden of HCV infection arises from its progression to chronic infection [3]. Approximately 74% of acutely infected patients progress to chronic infection [4] with 20% developing cirrhosis within 25 years and 25% of patients with cirrhosis developing hepatocellular carcinoma and decompensated liver disease [5,6]. In Europe, HCV is now a leading cause of cirrhosis and primary liver cancer [7].

### People Who Inject Drugs and Hepatitis C Virus

Injection drug use is now one of the main routes of transmission of HCV globally, including Ireland, with an estimated 80% of new infections occurring among people who inject drugs (PWID) [8]. The number of HCV-positive cases among PWID is estimated globally at 10 million [9]. In Ireland, a study of anonymized data from the National Drug Treatment Reporting System in 1991-2014 estimated the total number of injectors as 16,382 up to 2014 with an estimated 56% chronically infected with HCV. After adjusting to account for injectors who had never shared injection equipment, it was estimated that 12,423 were infected with HCV with 9317 infections being chronic [10].

HCV infection is mostly asymptomatic in its early stages and the rate of progression is slow with manifest liver disease uncommon within the first 20 years of established infection [11]. As a result, without adequate screening measures, there is marked potential for an elevated burden of disease among an aging population of former or current PWID. Furthermore, injection drug use is associated with high levels of problem alcohol use as well as other factors associated with adverse outcomes [12,13]. In Ireland, a national cross-sectional study reported that 35% of patients attending general practitioners (in Ireland “general practitioner” is the term used for a primary care physician) for opioid substitution treatment also had problem alcohol use [14], whereas findings from a subsequent qualitative study highlighted the need for an educational intervention to address this problem in primary care [15]. Alcohol use disorders accelerate the progression to fibrosis and associated complications; therefore, it is important that alcohol use is addressed within HCV care [16,17]. Guidelines issued by the World Health Organization and the Health Services Executive in Ireland outline the best practices for HCV care, including addressing problem alcohol use [18,19].

In Europe, primary care is increasingly involved in providing continuing care, including opioid substitution treatment, for PWID. Ireland has developed a model of care for delivering opioid substitution treatment, which acknowledges the central role a general practitioner plays in delivering care [20]. Methadone is a long-acting synthetic opioid with documented safety, efficacy, and effectiveness prescribed for opioid substitution treatment [21]. To prescribe methadone in Ireland, general practitioners are subjected to clinical audit and must complete special training with general practitioners providing methadone treatment for ≥15 patients subject to more regular audit and advanced training. General practitioners who prescribe methadone for <15 patients are referred to as “level 1” general practitioners and those prescribing for ≥15 as “level 2” general practitioners [14]. Patients on opioid substitution treatment attend their general practitioner regularly (at least weekly), which offers the general practitioner a chance to monitor for drug and alcohol use and provide education around harm reduction strategies. However, general practitioners have highlighted challenges in providing opioid substitution treatment, including a fear of violence, alienating other patients, lack of knowledge, lack of community supports, and personal reasons such as a lack of empathy with addicts and discomfort with the topic of addiction [20].

### Primary Care and Hepatitis C Virus

The important role of primary care in HCV screening and the provision of complex interventions tailored to the needs of PWID have been highlighted previously [22-25]. Although primary care can be a challenging place to deliver interventions given time and resource constraints, the mobility of new HCV diagnostic modalities, including liver stiffness measurement by transient elastography (FibroScan), means it can be transported to community sites to access patients’ disease severity. Furthermore, utilizing an HCV liaison nurse in primary care settings can enhance HCV assessment, reduce patient-identified barriers, target therapy, and enable the triage of patients for more immediate care [26-28]. Trials of integrated models of care for HCV treatment (ie, incorporating treatment for mental health issues and substance abuse) in primary health care settings have shown promising results for improving outcomes among PWID [29-31].

The recent emergence of direct-acting antiviral (DAA) treatments has dramatically improved treatment outcomes for patients with HCV; these treatments have shown >90% efficacy in achieving the sustained virological response. The utilization of these new therapeutic agents, combined with improved testing, linkage to care, and adherence to treatment, shows promise for counteracting the expected rise in the future disease burden [32].

Irish and international strategies, plans, and guidelines have prioritized the provision of new pharmaceutical regimens for patients with the greatest clinical need initially, seeking to balance the high cost of these drugs at present with a view for the wider implementation of these drug regimens in the future [33,34]. In addition, historically HCV screening has been problematic with blood tests and liver biopsy being the standard approach to assess the need for treatment causing many patients to avoid HCV care because of the perceived dangers associated with liver biopsy [35].

A cross-sectional survey of patients attending general practice (in Ireland “general practice” is the name given to the service provided by general practitioners) in the Eastern region of Ireland reported HCV testing rates of 34% and the HCV prevalence rate of 73% among those tested [36]; this low rate of screening for HCV in general practice has also been reported in other health care systems [37,38].

### Aims

This study aims to describe the current management of HCV among patients on opioid substitution treatment attending general practice in Ireland in light of the current guidelines aimed at scaling up interventions to reduce chronic HCV infection and associated mortality [39,40]. The data presented in this paper are the baseline data from the Dublin site of the Heplink study [41], which is a feasibility study focusing specifically on developing complex interventions to enhance community-based HCV treatment and improve HCV care pathway in primary and secondary care. The Heplink study is one component of the Hepcare Europe project [41,42], an EU-supported service innovation project and feasibility study at 4 European sites (Dublin, London, Seville, and Bucharest) to develop, implement, and evaluate interventions to enhance the identification and treatment of HCV among PWID.

## Methods

### Study Design

The research examined the current HCV care practice using a nonprobability sample of patients attending general practices across North Dublin for opioid substitution treatment. Data were collected from clinical records as part of a feasibility study of a complex intervention to enhance HCV care. Outcomes will be discussed relating to blood-borne virus care and problem alcohol use.

The study sites were 14 opioid substitution treatment-prescribing general practices located in Dublin’s north inner city. In Ireland, currently, there are 2 types of settings in which opioid substitution treatment is delivered in the community—specialist addiction clinics and general practice. All patients receiving opioid substitution treatment are registered on the Central Treatment List. “Level 1” general practitioners are responsible for the treatment of stabilized opiate-dependent persons referred from specialist addiction clinics or from “level 2” general practitioners. Practice as a “level 1” general practitioner requires the completion of a recognized training program conducted by the Irish College of General Practitioners and regular educational updates. General practitioners are audited by the Irish College of General Practitioners through Health Services Executive Audit Committee. “Level 1” general practitioners can treat up to a maximum of 15 patients. A “level 2” general practitioner who has undergone additional training can initiate opioid substitution treatment and prescribe for a higher number of patients (up to a maximum of 35 patients or a maximum of 50 in a partnership with ≥2 doctors in their practice [43]). As of August 31, 2016, there were 9652 patients receiving treatment for opiate use in Ireland (excluding prisons), which included 4150 patients being treated by 350 general practitioners in the community [44].

We recruited 14 opioid substitution treatment-prescribing general practices in North Dublin from the professional networks and databases of the research team. Practices were eligible to participate if they were registered to prescribe opioid substitution treatment and were located within the Mater Misericordiae University Hospital (MMUH) catchment area. We wrote to all general practices (n=63) within the referral area of the MMUH Infectious Diseases Department and specified this inclusion criterion in our letter of invitation. We only invited expressions of interest to be returned if practices met this inclusion criterion. A standardized nonprobability sampling framework was used to identify 10 patients from each practice to participate in the study. Standardized nonprobability sampling is commonly used in feasibility studies in which samples are selected on the basis of the subjective judgment of the researcher rather than random selection (ie, probabilistic methods), which is the cornerstone of probability sampling techniques. Based on the recommendations for good practice in feasibility studies [45] and our previous feasibility studies among PWID [46,47] we estimated that 140 patients (attending 14 general practices) would be adequate to calculate the actual recruitment and retention rates (ie, feasibility) and provide data on the acceptability of study processes and outcome measures to inform a future definitive intervention trial. Patients were eligible to participate if they were aged at least 18 years, were on opioid substitution treatment, and attended the practice for any reason during the recruitment period. The researchers instructed participating general practitioners to recruit consecutively presenting patients who were eligible until they had attained a quota of 10.

General practitioners provided eligible patients with a verbal explanation of the study and a written information leaflet outlining the study’s purpose, procedures, and how the findings would be utilized. Patients who were interested in participating were asked to sign a consent form, which was witnessed by a general practitioner or a member of the research team. Although the initial approach to participate was from a general practitioner, recruitment was facilitated by a member of the research team being “on site” (where feasible) to support the practice during the recruitment phase and answer any questions that the potential participants might have. Although practices were instructed to recruit 10 eligible patients, some practices recruited <10 because they had a smaller number of opioid substitution treatment patients, and some practices recruited >10 patients. Ethical approval for the study was received from the MMUH Research Ethics Committee.

### Data Collection and Analysis

The clinical records of participating patients were examined by a member of the research team prior to the implementation of the “Heplink” intervention. Baseline data were extracted on demographic characteristics, care processes and outcomes in relation to HCV and other blood-borne viruses (BBVs), urinalysis test results, problem alcohol use, chronic illness, and health service utilization. Variables were chosen to provide data on patients’ present and historical HCV care as well as other BBVs (hepatitis B virus, HBV, and HIV). In addition, data were collected on service utilization (emergency department visits and accessing general practice out-of-office hours service) to identify high utilizers and access to care. The baseline data collection instrument was expanded midway through the study to collect additional information on BBV care from the 7 practices (n=60 patients) who participated in the study thereafter. It is our intention to collect this additional baseline information from the other 7 practices when we return to all practices to collect the 6-month postintervention follow-up data and the baseline information will be reported in our pre-post intervention data manuscript which is in preparation. Furthermore, means, frequencies, and percentages were calculated using IBM SPSS Statistics Software version 24.

### Blood-Borne Virus Care

Lifetime and past 12-month data on HCV care were extracted from each clinical record, including the following: HCV antibody testing (yes or no) and status (positive or negative); whether a patient had been referred to a hepatology or infectious diseases specialist (yes or no); had attended a hepatology or infectious diseases specialist (yes or no); been assessed by Fibroscan (yes or no); Fibroscan scores (kPa); and initiated HCV treatment (yes or no). In addition, lifetime and past 12-month data in relation to other BBVs were also extracted, including the following: HIV antibody and hepatitis B surface antigen (HbsAg) or hepatitis B core antibody (anti-HBc) testing (yes or no) and status (positive or negative) and whether a patient had received any dose of HBV immunization (yes or no). Additional data on BBV care were extracted on a subset of 7 practices (n=60 patients), including the following: HCV RNA and antigen (Ag) testing (yes or no) and status (positive or negative); whether HCV treatment had been completed (yes or no); and whether a patient had received any dose of hepatitis A virus immunization (yes or no). Furthermore, lifetime and past 12-month data in relation to these variables were collected.

### Problem Drug and Alcohol Use

Data extracted from clinical records included results of the last urine drug test, that is, whether positive or negative for metabolites of illicit drugs and non-prescribed benzodiazepines. In addition, clinical records were reviewed to determine whether a general practitioner, in the past 12 months, had screened or discussed alcohol use (yes or no); conducted a brief intervention (yes or no); and referred a patient to specialized treatment (yes or no).

### Chronic Illness and Health Service Utilization

Data on the presence of chronic illnesses (yes or no) and whether a patient had any emergency department visits in the past month (yes or no) or general practice out of hours visits in the past month (yes or no) were extracted.

## Results

### Sample Characteristics

In this study, 14 general practices and 135 patients were recruited. Although 7 practices were “level 1” prescribers, 7 practices were “level 2” prescribers. The baseline data were collected from the clinical records of 134 patients; of these, 71.6% (96/134) were males, and the mean age of the sample was 43 (range 27-71; SD 7.6) years. In addition, 37.3% (50/134) patients’ most recent urine sample had tested positive for metabolites of nonprescribed drugs of abuse. Of note, 36.6% (49/134) had at least one chronic illness documented in their clinical record. Although 2 patients (1/134, 0.7%) had visited an emergency department in the past month. None of the patients had attended a general practice out of hours service in the past month.

### Screening for Blood-Borne Viruses

In this study, 94.8% (127/134) patients had been tested for anti-HCV antibody in their lifetime; of those, 77.9% (99/127) tested positive. In the 12-month period before the study, 23.9% patients (32/134) had received an anti-HCV antibody test and 23 (23/32, 72%) of those tested positive.

Next, 112 of 134 (83.6%) patients had received an HIV antibody test in their lifetime; of those, 7 (6.3%) tested HIV positive. Furthermore, 34 (25.4%) patients had been tested for the HIV antibody in the previous 12 months and 1 (1/34, 3%) tested positive.

Furthermore, 66.4% (89/134) patients had been tested for HBV in their lifetime, and 7 (7/89, 8%) of those tested were positive; 22.4% (30/134) had been tested in the previous 12 months, with one (1/30, 3%) testing positive (Table 1).

### Immunization Against Other Hepatotropic Viruses

Of 134 patients, 48.5% (65/134) had received at least one dose of HBV immunization in their lifetime, and 8.2% (11/134) had received at least one dose in the 12 months prior to the study. Additional data collected from 7 practices (n=60 patients) showed evidence that 28% (17/60) patients had received at least one dose of Hepatitis A virus immunization in their lifetime with 2% (1/60) receiving the vaccine in the past 12 months (Table 2).

### Subsequent Care of Anti-Hepatitis C Virus Antibody-Positive Patients

Of 99 patients known to be anti-HCV antibody-positive, 17% (17/99) patients had undergone a Fibroscan in their lifetime, and 5% (5/99) had undergone a Fibroscan in the previous 12 months. Fibroscan scores were available for 16 patients; the mean score was 7.0 kPa (SD 3.4; range 0.1-16.9). In addition, 20% (20/99) patients had initiated HCV treatment in their lifetime, and 3% (3/99) had initiated treatment in the past 12 months.

**Table 1:** Blood-borne virus screening and infection status.

Outcome	Total (n=134), n (%)
HCV^a^ Ab^b^ test—lifetime	127 (94.8)
HCV Ab positive—lifetime	99 (73.9)
HCV Ab test—past year	32 (23.9)
HCV Ab positive—past year	23 (17.2)
HIV Ab test—lifetime	112 (83.6)
HIV Ab positive—lifetime	7 (5.2)
HIV Ab test—past year	34 (25.4)
HIV Ab positive—past year	1 (0.7)
Anti-HBc^c^ or BsAg^d^ test—lifetime	89 (66.4)
Anti-HBc or HBsAg positive—lifetime	7 (5.2)
Anti-HBc or HBsAg test—past year	30 (22.4)
Anti-HBc or HBsAg positive—past year	1 (0.7)

^a^HCV: hepatitis C virus.

^b^Ab: antibody.

^c^anti-HBc: hepatitis B core antibody.

^d^HBsAg: hepatitis B surface antigen.

**Table 2:** The hepatitis A and B immunization status.

Outcome	Total, n (%)
Any HBV^a^ immunization dose lifetime^b^	65 (48.5)
Any HBV immunization dose past year^b^	11 (8.2)
Any HAV^c^ immunization dose lifetime^d^	17 (28.3)
Any HAV immunization dose past year^d^	1 (1.7)

^a^HBV: hepatitis B virus.

^b^n=134.

^c^HAV: hepatitis A virus.

^d^n=60.

**Table 3:** The management of anti-HCV antibody-positive patients.

Outcome	Total, n (%)
Fibroscanned—lifetime^a^	17 (17)
Fibroscanned—past year^a^	5 (5)
Initiated HCV^b^ treatment—lifetime^a^	20 (20)
Initiated HCV treatment past year^a^	3 (3)
HCV Ag^c^ or RNA test—lifetime^d^	36 (76)
HCV Ag or RNA positive—lifetime^d^	22 (46)
HCV Ag or RNA test—past year^d^	5 (10)
HCV Ag or RNA positive—past year^d^	2 (4)

^a^n=99.

^b^HCV: hepatitis C virus.

^c^Ag: antigen.

^d^n=47.

**Table 4:** Screening and intervention for problem alcohol use in the past year.

Outcome	Total (n=134), n (%)
Alcohol screening past year	41 (30.6)
Alcohol brief intervention past year	8 (6.0)
Referred to specialist addiction or alcohol treatment service past year	3 (2.2)

Additional data collected from 7 (n=60 patients) of the 14 practices indicated that among 47 patients who tested HCV antibody positive in these practices, 77% (36/47) had received confirmatory HCV RNA or Ag testing in their lifetime with 47% (22/47) testing positive and 11% (5/47) had received RNA or Ag testing in the previous 12 months with 4% (2/47) testing positive (Table 3).

### Alcohol Screening and Brief Intervention

Of 134 patients, 41 patients (30.6%) were asked about their alcohol use by their general practitioner in the 12-month period prior to the study; 6.0% (8/134) had received a brief intervention (ie, a structured discussion around alcohol harms), and 2.2% (3/134) had been referred to a specialist addiction or alcohol treatment service in the same 12-month period (Table 4).

## Discussion

### Summary

Our findings (Table 5) suggest that although the testing rates for HCV among PWIDs attending general practice for opioid substitution treatment have increased since 2003, access to further assessment and antiviral treatment remains a challenge. Although 94.8% (127/134) of patients had been tested for anti-HCV antibody in their lifetime (of whom 99/127, 77.9% tested positive), only 17.2% (23/134) had had a Fibroscan and 20.2% (27/134) had initiated antiviral therapy. The fact that only 20.2% of patients have commenced antiviral therapy reflects the current high cost of antiviral drugs in Ireland, which prohibits the number of patients who can access treatment. Because the cost of antiviral drugs is predicted to decrease considerably over the coming years, the number accessing antiviral therapy will increase. In addition, it is a concern that 37.3% (50/134) of patients’ most recent urine sample had tested positive for metabolites of nonprescribed drugs of abuse; this highlights the need for general practitioners to engage in continued harm reduction education with this cohort of patients. The low immunization rates recorded (48.5%) may be attributed to immunization being carried out elsewhere and therefore not recorded in patients’ general practice records. The low screening rates for alcohol use (41/134, 30.6%) are similar to control group rates in a recent study examining screening and brief intervention for alcohol in general practice in Ireland [47] and highlight the need for more general practitioner training.

### Comparison With Existing Literature

In this study, 94.8% (127/134) of patients had been tested for anti-HCV antibody in their lifetime with 77.9% (99/127) testing positive; this shows an increase in the number of patients being tested for anti-HCV antibody compared with data collected in 2003 in a study examining HCV among opioid substitution treatment patients in primary care in Ireland (69%; Table 5) [48]. This increase in testing for the anti-HCV antibody is to be welcomed and reflects the increase in HCV outreach programs and better education of general practitioners in HCV care since the introduction of DAA drugs. The percentage of patients testing positive for HCV is higher than that reported in previous studies on opioid substitution treatment patients in primary care in Ireland [14,48,49] (Table 5) and higher than that in an at-risk cohort from the HepCAT study in primary care in the United States [50]. Furthermore, the percentage of patients testing HCV positive is considerably higher than available data on the prevalence of HCV among injection drug users in Ireland (41.5%) from the European Monitoring Centre for Drugs and Drug Addiction [51].

In this study, 6.3% (7/112) of patients tested positive for HIV, and 8% (7/89) tested positive for HBV (anti-HBc or HBsAg); these rates are lower for HIV but higher than the HBV data reported by Cullen et al (HIV 10%; HBV 4%) [48]. Compared with a more recent study by Klimas et al among opioid substitution treatment patients in primary care in Ireland, the rate for HIV is similar, but that for HBV is higher (HIV 5.7%; HBV 2.7%; Table 5) [49].

In addition, 30.6% of patients had been asked about their alcohol use by their general practitioner in the 12-month period prior to the study, 6.0% had received an alcohol brief intervention, and 2.2% had been referred to a specialist addiction or alcohol treatment service. Although these data show an improvement in screening, brief intervention, and referral to treatment for alcohol compared with the baseline data from a recent alcohol intervention study among opioid substitution treatment patients in primary care in Ireland [49,52], alcohol screening and brief intervention should be systematically performed in this cohort [53].

### Limitations

Limitations of this study include the use of a nonprobability sampling strategy; although this results in a lower level of generalizability of research findings and inability to calculate CIs and margins of error, it is an appropriate sampling strategy to use for populations of PWID [54] and when conducting a feasibility study in which lower sample sizes make probability sampling impractical [55]. In addition, potential bias and lack of generalizability may occur from general practitioners who are more motivated and enthusiastic about the issue under study being overrepresented among those recruited. Owing to their interest in the issue, general practitioners who self-selected to the study may be providing better HCV care to their patients than the wider general practitioner population, leading to higher screening rates being detected in the clinical records of participating patients.

**Table 5:** The blood-borne virus status among patients attending general practice in Ireland for opioid substitution therapy—comparison of 2017, 2013, 2003, and 1999.

Variable	1999	2003	2013	2017
Age (y)	28	32.2	40.9	43
Gender (male), n (%)	409 (72)	141 (72)	69 (65)	96 (72)
Tested for anti-HCV^a^ (lifetime), n (%)	380 (67)^b^	151 (77)	104 (99)^c^	127 (95)
HCV positive, n (%)	276 (73)^d^	104 (69)	54 (51)	99 (78)
Tested for HIV (lifetime), n (%)	326 (57)	135 (69)	103 (98.2)^c^	112 (84)
HIV positive, n (%)	27 (8)	14 (10)	6 (5)	7 (6)
Tested for HBV^e^ (anti-HBc^f^ or HBsAg^g^), n (%)	316 (55)	118 (60)	88 (83)	89 (66)
HBV positive (anti-HBc or HBsAg), n (%)	43 (14)	5 (4)	3 (2)	7 (8)

^a^HCV: hepatitis C virus.

^b^113 of 380 was self-reported data.

^c^Self-reported data.

^d^75 of 276 was self-reported data.

^e^HBV: hepatitis B virus.

^f^HBc: hepatitis B core antibody.

^g^HBsAg, hepatitis B surface antigen.

Furthermore, although only data on patients who consented to a researcher having access to their clinical records were collected, consent bias is likely to be minimal, particularly given the high proportion of those asked who provided consent. In addition, owing to the complex needs of PWID, many will have previously attended a different general practitioner(s) during their lifetime. Therefore, it is unlikely that practice clinical records can accurately capture “lifetime” blood-borne screening. The accuracy of practice clinical records can often be problematic in primary care research because of data not being updated or recorded consistently. However, the rich information contained in patients’ clinical records offers considerable potential for research purposes, and our research team aimed to minimize inaccuracies by asking the general practitioner to review the extracted data. Furthermore, data from clinical records do not capture important variables such as socioeconomic variables (income and education) and psychosocial variables (levels of social support), and a data instrument to collect these variables should be added to future research in this area. In addition, the “chronic illness” variable used was dichotomous and therefore hides a lot of information, and this should be addressed for future studies. A further limitation is the comparison data presented in Table 5. There were wide variations in sample sizes between the datasets with some data being self-reported. As such, the conclusions drawn from comparisons of these datasets must be treated with some caution.

Despite these sources of potential bias, the general practitioners and patients who participated in the study were comparable in their profile to other studies from Ireland [48]. Furthermore, this is the first study in Ireland to examine the feasibility of integrated HCV care among problem drug users attending primary care. It will provide positive public health implications with key data to enhance the scientific understanding of interventions that prevent risk behaviors, inform policy and service development, and contribute to the health and social gain locally and internationally. Furthermore, it is the intention of the research team to complement this urban-living population study with similar studies in rural-living populations in Ireland. If the intervention proves feasible, we also intend to scale up the study to other geographical areas recruiting larger sample sizes.

### Conclusions

The advent of highly effective DAAs have made eradicating hepatitis C possible, but for this to occur, health care systems must address the complex and wide-ranging difficulties associated with effective HCV screening, assessment, and treatment in the community. With general practice and primary care playing an increased role in HCV care, this study suggests that the development and evaluation of real-world clinical solutions, which support patients from diagnosis to completing treatment, are a priority.

## Abbreviations

Ag: antigen
BBV: blood-borne virus
DAA: direct-acting antiviral
EU: European Union
HAV: hepatitis A virus
HBc: hepatitis B core antibody
HBsAg: hepatitis B surface antigen
HBV: hepatitis B virus
HCV: hepatitis C virus
MMUH: Mater Misericordiae University Hospital
PWID: people who inject drugs
